# Supplementary material for: A Rapid, Sensitive, Low-Cost Assay for Detecting Hydrogenotrophic Methanogens in Anaerobic Digesters Using Loop-Mediated Isothermal Amplification
Source: Microorganisms. 2020 May 15;8(5):740. doi: 10.3390/microorganisms8050740 (PMC7284894; doi:10.3390/microorganisms8050740)
Supplement: Supplementary file 1 [file microorganisms-08-00740-s001.pdf]

Table S1. Sanger sequencing results of the PCR products for aEF-2 genes of selected positive controls.

Sample ID#	Sequence 5'-3'	Primer used	BlastN results
**PCR4_Mco**	atGGnAcaCaTGaACaAATTAGAAACATGGGTATCTGTGCTCACATTGACCACGGTAAAACCACATTATCAGACAACTTGCTTGCAGGAGCAGGTATGATCTCCAAAGAATTAGCTGGAGACCAGCTTGCACTTGACTTCGATGAAGAAGAAGCTGCAAGAGGTATCACAATCTACGCTGCAAACGTTTCAATGGTACACGAATACAGCGGAAAAGAGTACTTAATCAACTTAATCGATACCCCTGGTCACGTTGACTTTGGTGGTGACGTTACAAGAGCAATGAGA	F3_aEF2_Mco<br>5'ATGGGAAGAAGAGCAAAAATG3'	*Methanococcus maripaludis*
**PCR5_Mcu**	gnnGTGacCCGCGCCaTGCGcnGCGGTGnGACGGCGCCGTCGTCGTGGTGGACGCGGTCGAGGGGACCATGCCCCAGACGGAGACGGTGCTCCGGCAGGCCTTgAagGAagGCGTCCGCCCGGTTCTCTTCATCAACAAGGTCGACCGGCTGGTCAACGAGCTGAAGGTGGACGAGCAGGAGATGCAGATCCGCCTCGCGAAGGTGATCGACAAGGTCAACAAGCTGATCAAGGGCATGAACgAaaAA	F3_aEF2_Mcu 5'<br>TAYCTBATCAACATGATYGAT3'	*Methanoculleus marisnigri*
**PCR6_Mth**	ggtntaGTGgCCcaCATTGACcaCGGGAAAACaACACTTTcaGACAACCTCCTGGCTGGTGCAGGGaTGATCTCCGCCGAACTGGCCGGGGATCAGCGCTTCCTTGACTTCGACGAACAGGAACAGGCAAGGGGTATCACCATTGACGCTGCAAACGTCTCAATGGTCCACTCCTACGAGGGAAATGAATACCTCATAAACCTCATCGACACACCAGGTCACGTTGACTTCGGGGGGGACGTTACAAGGGCCATGAGGGCCGTGGACGGTGCAGTTGTTGTCGTATGCGCCGTTGAGGnnaTAAt	F3_aEF2_Mth<br>5'ATTAAGGAGCTCATGTACCA3'	*Methanothermobacter thermauthotrophicus*

# - see Figure 1

[redacted]

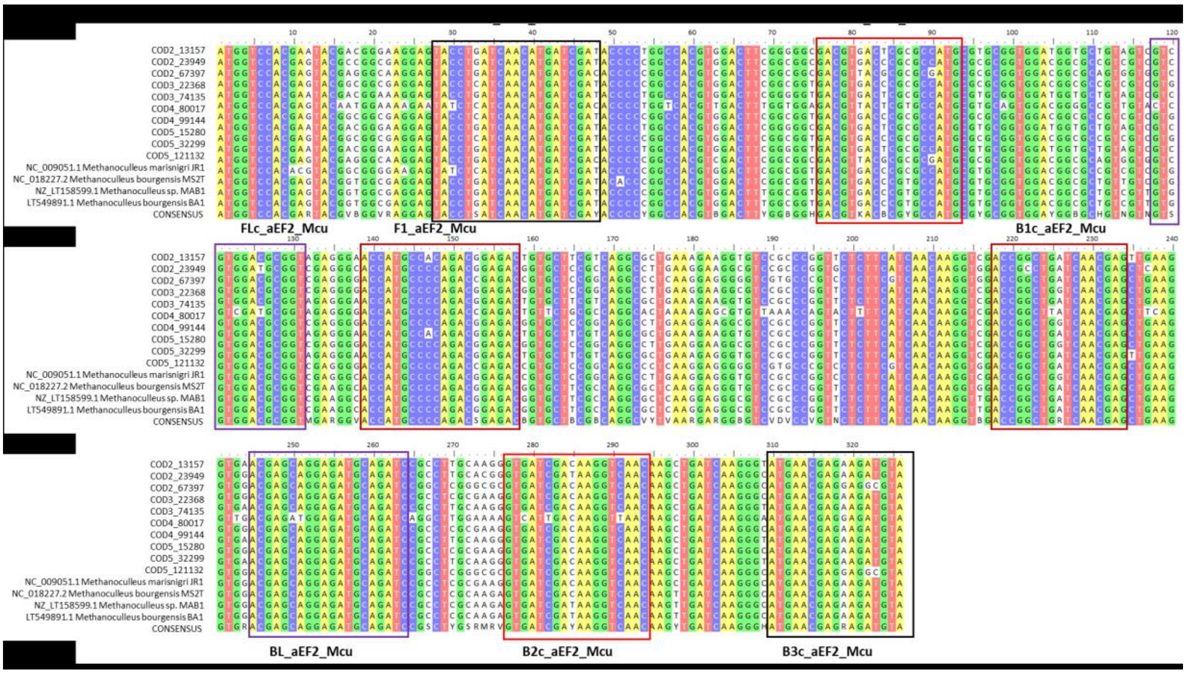

[redacted]

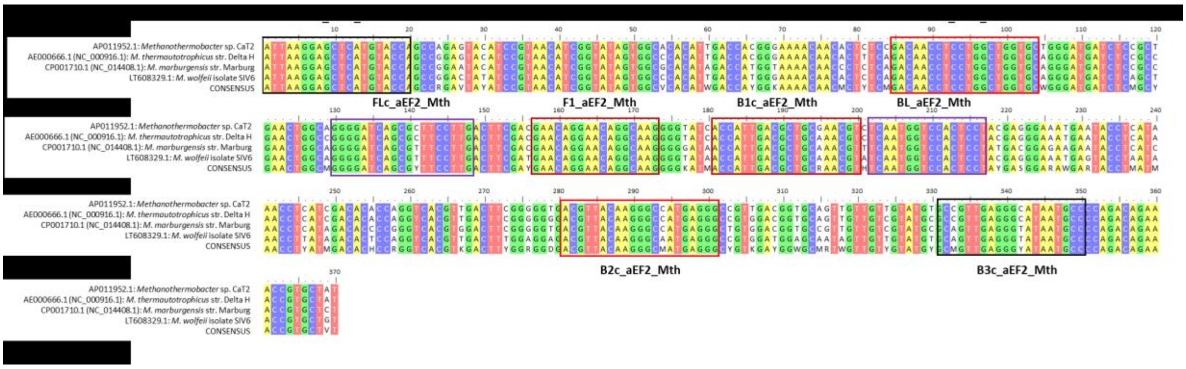

[redacted]

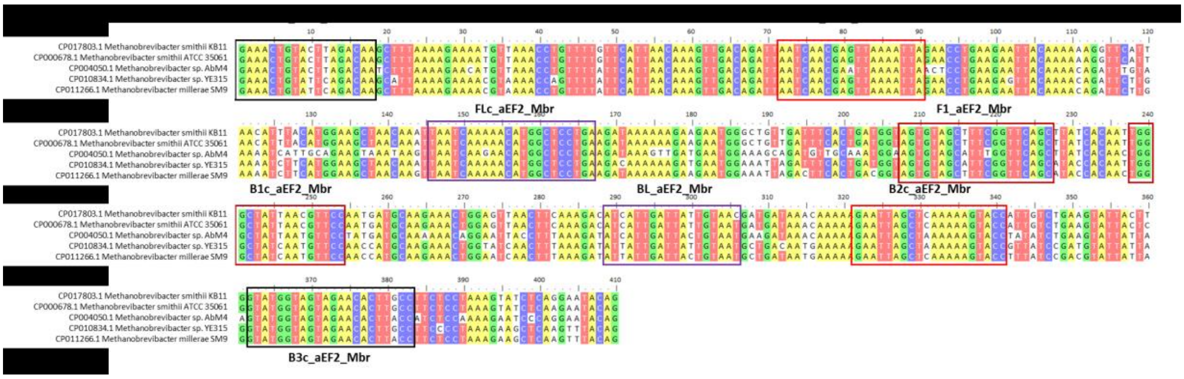

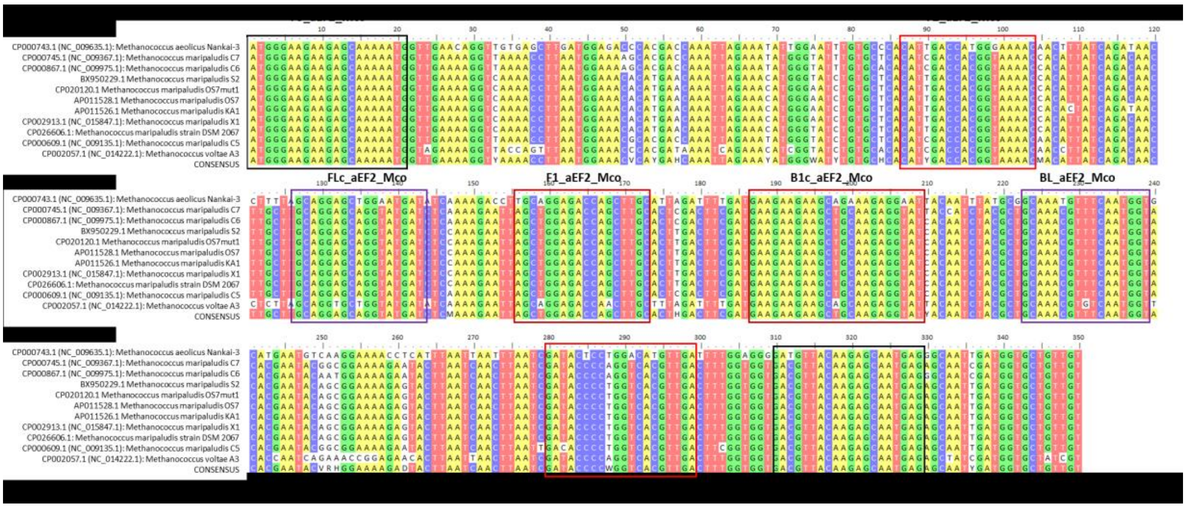

Figure S1. Alignments of aEF-2 genes from selected genera of hydrogenotrophic methanogens used to design the LAMP primers. COD designated samples originate from a study published by (Tao et al. 2019). (a) *Methanoculleus* sp., (b) *Methanothermobacter* sp., (c) *Methanobrevibacter* sp., (d) *Methanococcus* sp.
